# Health inequalities, physician citizens and professional medical associations: an Australian case study

**DOI:** 10.1186/1741-7015-5-23

**Published:** 2007-08-13

**Authors:** John Furler, Elizabeth Harris, Mark Harris, Lucio Naccarella, Doris Young, Teri Snowdon

**Affiliations:** 1 Department of General Practice, University of Melbourne, Australia; 2 Centre for Primary Health Care and Equity, University of New South Wales, Australia; 3 Royal Australian College of General Practitioners, South Melbourne, Victoria, Australia

## Abstract

**Background:**

As socioeconomic health inequalities persist and widen, the health effects of adversity are a constant presence in the daily work of physicians. Gruen and colleagues suggest that, in responding to important population health issues such as this, defining those areas of professional obligation in contrast to professional aspiration should be on the basis of evidence and feasibility. Drawing this line between obligation and aspiration is a part of the work of professional medical colleges and associations, and in doing so they must respond to members as well as a range of other interest groups. Our aim was to explore the usefulness of Gruen's model of physician responsibility in defining how professional medical colleges and associations should lead the profession in responding to socioeconomic health inequalities.

**Methods:**

We report a case study of how the Royal Australian College of General Practitioners is responding to the issue of health inequalities through its work. We undertook a consultation (80 interviews with stakeholders internal and external to the College and two focus groups with general practitioners) and program and policy review of core programs of College interest and responsibility: general practitioner training and setting of practice standards, as well as its work in public advocacy.

**Results:**

Some strategies within each of these College program areas were seen as legitimate professional obligations in responding to socioeconomic health inequality. However, other strategies, while potentially professional obligations within Gruen's model, were nevertheless contested. The key difference between these lay in different moral orientations. Actions where agreement existed were based on an ethos of care and compassion. Actions that were contested were based on an ethos of justice and human rights.

**Conclusion:**

Colleges and professional medical associations have a role in explicitly leading a debate about values, engaging both external stakeholder and practicing member constituencies. This is an important and necessary step in defining an agreed role for the profession in addressing health inequalities.

## Background

Confronting the health effects of social inequality is part of the daily world of medical practitioners. General practitioners (GPs) in particular are constant witnesses to the important effects that adverse social and economic circumstances have on the health of their patients and their communities [1-6]. Yet responding effectively to this challenge has been a vexed issue for the profession as it struggles to define a role that is realistic and broadly accepted by practitioners [7,9-12]

**Figure 1:**
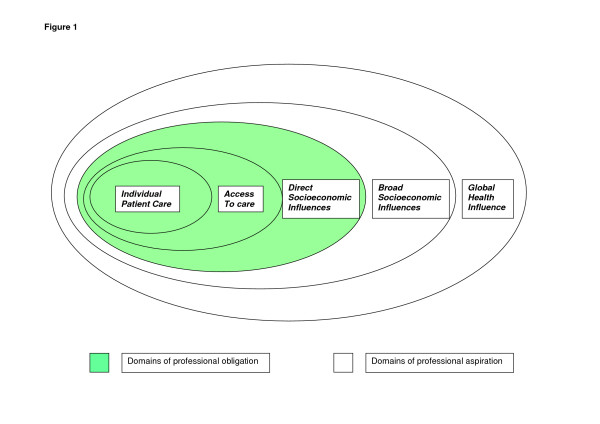
**Model of physician responsibility in relation to influences on health**. Source: Gruen RL, Pearson SD, Brennan TA: **Physician-citizens – public roles and professional obligations. ***JAMA *2004, **291:**94–98.

Gruen and colleagues suggest that defining an appropriate role for physicians in relation to such public health issues should be undertaken on the basis of evidence and feasibility [13]. They suggest five domains where there is potentially a role for physicians (Figure 1). Three of these domains, where the link between policy, action and health is well-established, they describe as domains of professional obligation. These include the provision of high quality patient care, ensuring access to health care and addressing direct socioeconomic influences on health such as smoking, injury and availability of clean needles for drug users. Two other domains, where the causal links with health are less well understood and the feasibility of effective action by the medical profession is less obvious, are described as domains of professional aspiration. These include the broader social and global determinants of health, such as the impact of income inequality on health and wellbeing. They argue that their model is flexible and that, as the strength of evidence changes or accrues of a relationship between socioeconomic factors and health and feasibility of action becomes clearer, then the line between professional obligation (with responsibility to intervene) and aspiration may shift.

Professional medical colleges and associations have an important role to play in defining the line between professional obligation and aspiration. Yet this is increasingly difficult in a rapidly changing environment. Traditionally, their roles have been to "perfect and protect the profession" [14]. However, competing economic, commercial and political agendas complicate this work. Armstrong [15] suggests that we are now seeing the "rise of an administrative elite, often grouped around the academy and professional colleges" who are balancing tensions between a wider community of external stakeholders on one hand and the interests of their members on the other. The concern of external stakeholders may be in mobilizing the credibility and expertise of the profession in collective activity on public health issues, while members are also concerned with the economic viability and autonomy of their practice.

In this paper, we describe the outcomes of a consultative process undertaken by the Royal Australian College of General Practitioners (referred to hereafter as the RACGP or the College) to examine the most effective ways for the College to respond to the important issue of health inequalities. The RACGP, with over 14500 members, is the largest single professional medical college in Australia. The College's mission is "To benefit our communities by ensuring high quality clinical practice, education, and research for Australian general practice, and supporting our current and future members in their pursuit of clinical excellence" in Australia [17]. The College is also committed to "advocating for equity and enhanced access to General Practice for all people". This paper focuses on two major areas of activity within the RACGP (its role in setting standards for training of general practitioners and in setting practice standards that are used for accreditation of general practices) as well as the Colleges role in public advocacy. Our findings build on Gruen's model and increase its usefulness in health policy development in a real world setting and in addressing an important public health issue such as health inequalities.

## Methods

Our study ran from November 2000 to February 2002. Over this period we undertook a consultation involving multiple methods. We reviewed policy and program documents and undertook 80 semi-structured interviews (Table 1). Policy and program documents were selected as relevant to the education, training, standard setting and accreditation programs of the College. Interviews were conducted with key people internal and external to the College who had a capacity to effect change. In addition, we conducted two focus groups; one based in a metropolitan area and one in a rural setting. All of the focus group participants were practicing GPs and had at least some involvement in undergraduate or postgraduate GP training. Not all focus group members were members of the RACGP.

**Table 1:** Data sources

**Data source**	**No.**	**Descriptor/examples**
Program documents	10	Training program curriculum and learning companion, handbook, log book, Indigenous health training module, Standards for General Practice Manual
Policy documents	33	College position statements covering a range of areas (e.g. core professional issues and values statements, policies on domestic violence, health and the environment, evidence based medicine)
Internal College respondents	37	Program managers within the national office of the RACGP, chairs of national committees of the college, and leaders of state faculties of the college
External college respondents	43	Representatives of ADGP (the peak body of Divisions of GP in Australia)* and GPPAC (at the time the peak advisory body to the Federal Minister for Health), heads of academic Departments of General Practice, University Departments of Rural Health, consumer groups and a national PHC quality assurance program
Focus group participants	11	Urban and rural GP

*Divisions of General Practice are geographic organizations of GPs established in 1992 by the Government. They are local organizations that unite GPs and increase their capacity to work co-operatively with each other and other health providers.

Interviews consisted of a mixture of semi-structured open-ended questions and fixed response questions. The main focus was in defining the potential role of the College in addressing health inequalities within areas that were clearly part of its core business: the setting of standards for training and ongoing practice and practitioner accreditation. We also explored the advocacy role of the RACGP. Data analysis was through open coding and thematic analysis. This was undertaken separately by three of the authors (JF, EH, LN) to identify the breadth of issues emerging and consensus was reached on topics of emerging importance. We undertook iterative re-reading of data from across the interviews and focus groups within each of the program areas, seeking similarities, supportive and disconfirmatory evidence for identified themes. Emerging results were considered at sequential meetings of the project reference group. Findings from the thematic analysis were checked for validity through presentations made to staff and GP members at the national office and state faculties of the College. These meetings also included participants from the local University Department of General Practice, and representatives of Divisions of General Practice. The final report incorporated feedback from those meetings.

## Results

We found widespread acknowledgement that the College had an important and leading role to play in enhancing the way the profession addressed health inequalities through its work, consistent with the mission statement of the college. However, the way in which this should be undertaken was often contested. This was true even in areas that Gruen et al defined as areas of professional obligation, such as reducing financial barriers to improve access to care.

### Education and training

Most agreement about an appropriate role for the College in addressing health inequality was evident in the area of GP education and training, an area in which the College clearly has a mandate to act and capacity to have direct influence. At the time of the interviews the RACGP coordinated and provided all training to GPs nationally. In 2002, following a government review, an independent body was established to coordinate GP training through regional consortia [18]. Nevertheless, the RACGP retains control over standard setting for training, through its role in development of a national training curriculum and in maintaining the examination endpoint.

Consensus on how the College could assist in defining an appropriate role for the profession emerged from both program documents and interviews. The curriculum documents, for example, make many references to social inequality, both directly and indirectly. The GP is discussed as "...an agent of change in the improvement of health...in the community" and there is a stated commitment to developing trainees awareness of "issues of equity and access" [19]. There are specific curriculum modules addressing issues of social disadvantage including a module on Indigenous health. It is indirectly referred to in the Statements of Principle that training should be "community responsive". Importantly, this material within the curriculum exposes training GPs to the notion of advocacy.

In the interviews the key theme to emerge related to the importance of exposure to disadvantaged settings during training. This was seen as critical in shaping attitudes and an orientation to an extended role for practitioners outside the consulting room. It was felt that such exposure ought to be the norm in a training program. GPs, especially rural GPs, offered a number of practical ways in which this could be performed. These included placements in practices serving highly disadvantaged populations, GP supervisors acting as role models, encouraging trainees to undertake individual learning projects in relevant topic areas (such as homelessness) and the development of a community or practice socio-demographic profile. Providing such experiences through GP training can potentially occur in any practice, as most practices – even in advantaged areas – serve a socio-economically mixed patient group. Nevertheless, an analysis of the distribution of training practices at the time of the consultation showed a slight skew towards location in postcode areas of advantage rather than the reverse. This reinforced the view of some respondents that the program and policy documents were disconnected from real practice:

"These are great words but does it really sink in? There is too much ignorance out there." (GP Trainer)

There was also a concern that this was shifting the focus away from what ought to be the main concern of GP training, namely training doctors to provide high quality care to all patients:

"My personal feeling is that the College should be about training GPs, embryonic GPs and keeping its members as expert as possible with Continuing Medical Education (CME). I don't know that the College really needs to take on a role beyond that in term of society's wellbeing. It does that by providing good doctors in my belief." (Rural focus group GP)

There was hesitancy, mainly voiced by internal college respondents, about how prescriptive the College ought to be in mandating training exposure in particular disadvantaged settings. However, some external stakeholder respondents saw opportunities to further promote and develop such exposure through working with government to link training positions more directly to workforce recruitment and retention programs targeting disadvantaged areas of high need:

"The College ought to recognize that new graduates are a resource that ought to somehow be deployed to 'hot spots' where care provision is poor." (External interview respondent)

Thus, there was agreement that the College had an important role in training GPs to be sensitive to the needs of socio-economically disadvantaged communities and that training exposure was the best way to do this. What was contested was the extent to which this ought to be seen as obligation or aspiration.

### Practice standards, accreditation and quality of care programs

The RACGP is responsible for the development and setting of practice standards [20]. Accreditation of general practices (currently a voluntary process) against these standards is managed by a number of organisations external to the College. The College also manages a national program of quality assurance and continuing professional development (QA/CPD) for individual GPs, who participate on a three yearly cycle (again, currently a voluntary program). While we asked about practice standards and practitioner certification separately, respondents tended not to separate the two in their discussion.

The practice standards manual does contain standards relating to the way a practice ensures patients are able to access longer consultations and interpreters. There is also a standard that assesses the extent to which a practice has an up-to-date awareness of relevant health, social, community services in its area. The QA/CPD program has a stated principle of "responding to community needs" [21]. Individual GPs may develop learning plans that provide an opportunity to highlight how their individual quality improvement activities do this.

However, there was much less clarity or consensus on ways to extend the activities of the College in this area. The document review revealed a number of gaps and opportunities in this regard. For example, the standards manual contains no clinical outcome indicators at the practice level that might offer benchmarks against which to assess reach of care amongst subgroups of the practice population (the College has produced a number of guidelines for clinical care, but these have not been systematically applied in audit or accreditation). The standard on awareness of local community services makes no reference to how this might then be used in providing targeted care to patients from adverse or disadvantaged circumstances. Learning plans for linking QA activity to community need are not mandatory.

In the interviews and focus groups, while there was a high level of agreement that the quality of care provided by GPs should in theory be consistent and equitable, the focus was very much on the patients who present to the practice. Linking standards of practice, accreditation and quality of care to the notion of reach, implementation and uptake within the wider population the practice served was highly contested. For example, there was very little support for standards around ensuring financial accessibility to care at a practice level. In part this reflected practical and realistic issues such as the difficulties and sensitivities of identifying patients as "disadvantaged" and also concerns for the practice's own financial viability. A GP in the focus group expressed this:

"I actually think if we set a standard that says we will have equitable financial access, it will just kill my practice and hence it will have a significant impact on my community. So I think sort of the concept is simplistic ...and consider it insulting." (Focus group GP)

In the interviews many, especially external stakeholders, felt that setting practice standards focused on equity was an important area to explore, commenting that it was "a quality of care issue". However all of these activities were contested, particularly by internal college respondents and within the focus groups. Internal respondents particularly noted the difficulty in setting a standard in relation to a population health issue where the underlying determinants were seen to be outside the health sector. GPs in the focus groups were particularly resistant to the notion of setting standards addressing the coverage and reach of clinical care across disadvantaged groups at a practice level. They cited the burden associated with data collection and reporting, but primarily this was an ethical stance:

"...you can't practice medicine for different socioeconomic groups...practice medicine for the whole community." (Focus group GP)

It was suggested that if progress was to be made in developing an equity focus within standard setting and accreditation processes it would need to be a flexible and gradually evolving process that aimed to change the mindset of the profession as a whole, rather than specifically mandating particular activities:

"It would need to be a very broad principle and not too specific, for example simply a statement in the practice policy manual (about a commitment to address health inequality). It helps to move GPs along towards incorporating it in their thinking." (Internal College respondent)

### A public advocacy role of the College

Defining an appropriate advocacy role for the college emerged as an important issue. In the past, the College has taken on a public advocacy role in some areas beyond its traditional concerns with professional development. These have included, for example, the issuing of policy statements on Aboriginal reconciliation and the human rights of refugees and asylum seekers [22].

In the interviews and focus groups there was considerable discussion and disagreement about whether this was an appropriate role for the College. Almost all external stakeholders were strongly supportive of an important public advocacy role, seeing this as urgent and feasible. Within the college there was some support for prioritising this on the basis that the College could influence both Government and its members:

"In the future we need to be strong advocates. For example the government is pouring money into rural areas when urban deprivation receives no attention and the College needs to take a role in pointing this out." (Internal college respondent)

"It is a continual process of promoting a reorientation of practice. The College has a role in promoting the vision that GPs can make a difference and promoting the idea of fairness." (Internal college respondent)

Suggestions for ways to do this included linking with other medical colleges and associations, both nationally and internationally. However other internal respondents saw this as a low priority for the college, seeing the tension with member interests as too great:

"What do members want for their $900, do they pay us to just go off and develop beautiful policy? There must be a value in it, they want something back." (Internal college respondent)

## Discussion

How, then, does Gruen's model help us delineate an appropriate role for general practice as a profession in relation to the important public health issue of social inequalities in health? In particular, can a medical college help define an appropriate role for its members using the Gruen model? Certainly, within the domains of College activity we explored, a number of examples of action by the College can be identified that fit within Gruen's domains of professional obligation (Table 2). Ensuring appropriate exposure in training, for example, would improve the quality of GP-patient interaction when this involved patients from disadvantaged backgrounds. Considering social disadvantage when exploring a community profile or a training curriculum problem schema would build an awareness of barriers to accessing care and the way proximal social determinants affect the health of disadvantaged patients. However, there were actions within the area of professional obligation where considerable tension exists for members and the College. These particularly related to setting standards and mandating particular strategies (Table 2). There were also potential actions that fell in the area of aspiration where at least some agreement exists on an appropriate role for the college, such as the development of intercollegiate links to raise awareness of health inequities (Table 2).

**Table 2:** Gruen et al's domains matched to the RACGP case study

Domains of obligation and aspiration	*Obligation*			*Aspiration*
	
	Individual high quality patient care	Access to care	Direct socioeconomic influences	Broad and global socioeconomic influences
Examples of actions where broad agreement exists	**Ensure GP trainees exposed to work in disadvantaged communities**	**Ensure training includes opportunities to assess community need including local practice responses to unmet need**	**Include socio-economic context more prominently in the problem based curriculum** **Advocate for increased support for training practices in areas of disadvantage**	Formation of an intercollegiate group and a group within WONCA(World Organization of National Colleges and Academies of Family Medicine) to advocate on the link between socioeconomic disadvantage and ill-health
Examples of actions where disagreement exists	Mandating exposure to work in disadvantaged communities Develop standards on equity of reach of quality measures of clinical care within the known practice population	Advocate for practices to address financial barriers to accessing care Practices to assess unmet need in their area as a part of accreditation	Learning plans to be mandatory and linked to identified community need	

Bold type represent Gruen's areas of professional obligation where consensus exists for action.

The notion of a college or academy existing in tension raised by Armstrong helps to explain this. It also provides us with some suggestion as to a way forward. Armstrong suggests that the academy serves two masters. It leads the profession in claiming professional autonomy and the right to self regulation in a modern society through the adherence to evidence based practices by its members and a commitment to monitoring of quality and safety. However, it does so to some extent at the cost of reducing the clinical autonomy of its members. Regulation, monitoring and rational evidence based practice all demand adherence, compliance and reduced variability in the clinical work of its members. Armstrong calls this a "strategy of defending collective autonomy through restricting individual freedoms" [15].

The tensions manifest in our findings voiced by external stakeholders, internal College members and GPs in practice tend to reflect this difference between collective and individual autonomy. The positions adopted by respondents in this debate reflect important differences in underlying values and have implications for the sorts of strategies seen as appropriate in addressing health inequality. Exploring the values implicit in how people respond to the dilemma posed by Armstrong is important to understanding the tensions that emerged in our study and the implications for using Gruen et al's model.

### A question of values

Self et al [23] have described two different moral orientations evident within medicine. A "care orientation" is concerned with addressing vulnerability through support and attachment. A "justice orientation" addresses vulnerability through attention to fairness, rights and adherence to principles and standards. Using this framework, it is apparent that those areas where there is greatest consensus (Table 2) are driven by an ethos of care and compassion. In the examples given, the activities of the College and of its members are voluntary, aiming to develop sensitivity to and awareness of the needs of vulnerable and marginalised patients and communities. We identified possible strategies for the College that fall outside the area of professional obligation, yet which found some consensus in our consultation, for example, the broadly supported notion that the College link with other professional medical colleges internationally in raising awareness of health inequalities. Examples of this sort of work continue in the College's advocacy on environmental issues, nuclear disarmament, and on behalf of people with disability. Such strategies are supported possibly because they remain within this dominant moral orientation of care and compassion. However, there are actions that appear to fall within legitimate areas of professional obligation, yet are contested, possibly because they are driven by a different moral orientation (Table 2). Here actions are driven by an ethos of social justice and human rights. Setting standards on reach of quality care or mandating experience in work with disadvantaged communities is based on a notion that all people from all backgrounds have a right to a certain standard and accessibility of medical care. It implies not a discretionary activity of a practitioner guided by their moral values but a professional responsibility. It is this notion that seems to generate disagreement amongst the college respondents, external stakeholders and practicing GPs.

To some extent these moral orientations reflect the two directions of accountability that the college must serve, highlighted by Armstrong [15]. They also suggest different strategies for further action. An upwards accountability to Government and the wider community embraces the notion of a profession with a duty to serve the whole community, framed by notions of equity and human rights. In this context, the notion of obligation to a community or population rather than only to individuals who present for care is important. Targeted population approaches are needed to effectively identify local inequalities in health and health care. However, these may be contentious.

The downwards accountability to members needs to take account of the way practitioners embrace a compassionate, patient-centred, care orientation as a way of retaining a degree of individual clinical freedom and autonomy [15,24]. While this needs to be understood and respected, Colleges as leading professional bodies need to explore ways in which apparently legitimate areas of professional responsibility can be embraced by members without them feeling overwhelmed or further diminished in professional freedom. For example, while advocating for removing financial barriers to accessing care at a practice level, this needs to recognise the legitimate concerns of GPs not to have their incomes diminish. Safety nets have been negotiated for just this purpose in New Zealand where reduction of financial barriers for disadvantaged patients accessing general practice has been legislated [25]. Nevertheless, it remains true that balancing economic gain and security against extending the reach of and access to care does involve a tension between embedded value positions. Understanding and openly debating this may be important in resolving such tensions.

## Conclusion

Gruen's model is important as a starting point for negotiating a legitimate role for practitioners and the profession in engaging with public health issues of importance. Social inequalities in health are just such an issue. However, the model as it stands does not account for differences in interests between internal and external stakeholder perspectives, or for tensions between the interests of the professions leaders and practicing physicians.

Our study suggests that in drawing a boundary around core professional responsibilities, values need to be made more explicit and debated more widely. We believe that Colleges and professional medical associations have an important role to play in leading such a debate. Further, such a debate needs to make explicit the tensions between serving external stakeholders and members. In this way the College itself can help shift culture and thinking amongst both groups and make a vital contribution to efforts to tackle health inequalities.

## Competing interests

The authors declare that they have no competing interests.

## Authors' contributions

JF, EH, MH, LN and DY conceived the study and participated in its design, coordination, data analysis and drafting of the manuscript. JF and LN also conducted some of the interviews. TS participated in the study coordination, data analysis and helped to draft the manuscript. All authors read and approved the final manuscript.

## Pre-publication history

The pre-publication history for this paper can be accessed here:


